# Immunogenicity and safety of a novel MMR vaccine (live, freeze-dried) containing the Edmonston-Zagreb measles strain, the Hoshino mumps strain, and the RA 27/3 rubella strain: Results of a randomized, comparative, active controlled phase III clinical trial

**DOI:** 10.1080/21645515.2017.1302629

**Published:** 2017-03-31

**Authors:** Ashwani Sood, Monjori Mitra, Himanshu Arvind Joshi, Uma Siddhartha Nayak, Prashanth Siddaiah, T. Ramesh Babu, Samarendra Mahapatro, Jayesh Sanmukhani, Gaurav Gupta, Ravindra Mittal, Reinhard Glueck

**Affiliations:** a Department of Paediatrics, Indira Gandhi Medical College Shimla, Himachal Pradesh, India; b Institute of Child Health, Kolkata, West Bengal, India; c Department of Paediatrics, GMERS Medical College and General Hospital, Sola, Ahmedabad, Gujarat, India; d Department of Pediatrics, GMERS Medical College & General Hospital, Gotri, Vadodara, Gujarat, India; e Department of Pediatrics, Mysore Medical College and Research Institute and Associated Hospitals, Mysore, Karnataka, India; f Department of Pediatrics, Gandhi General Hospital, Musheerabad, Secunderabad, Telangana, India; g Department of Pediatrics, Hi-Tech Medical College & Hospital, Health Park, Pandara, Bhubaneswar, India; h Department of Clinical Research and Regulatory Affairs, Cadila Healthcare Limited, India; i Department of Virology and Biotechnology, Vaccine Technology Centre, Cadila Healthcare Limited, India; j Department of Clinical Research and Regulatory Affairs, Cadila Healthcare Limited, India; k Vaccine Technology Centre, Cadila Healthcare Limited, India

**Keywords:** Cadila Healthcare Limited, Hoshino mumps strain, novel vaccine, immunogenicity, MMR vaccine, measles, mumps, rubella, Serum Institute of India limited

## Abstract

This phase III clinical trial was conducted to evaluate the immunogenicity and safety of the single-dose and multi-dose formulations of a novel MMR vaccine (live, freeze-dried) developed by M/s Cadila Healthcare Limited, India (Cadila MMR vaccine), containing the Hoshino mumps strain, compared to that of an existing MMR vaccine (live, freeze-dried) developed by M/s Serum Institute of India Limited, India (Serum MMR vaccine). These two vaccines have similar measles and rubella strains, but different mumps strains (Hoshino in Cadila MMR vaccine, and L-Zagreb in Serum MMR vaccine). Three hundred and twenty-eight subjects of either sex, aged 15–18 months, were randomized in a 2:1 ratio to receive either the Cadila or Serum MMR vaccine. Immunogenicity assessments (IgG antibodies against measles, mumps, and rubella viruses) were done at baseline and 42 d after vaccination. Solicited (local and systemic) and unsolicited adverse events were recorded for up to 42 d following vaccination. The Cadila MMR vaccine was found to be non-inferior to the Serum MMR vaccine in terms of end-of-study proportion of subjects seropositive for anti-measles antibodies (100.0% in both groups), anti-mumps antibodies (94.5% vs. 94.0%), and anti-rubella antibodies (95.5% vs. 91.0%). Both vaccines were well tolerated by all study participants; the most common adverse event reported in both groups was fever, followed by rash. The results of this phase III clinical trial show that the novel Cadila MMR vaccine is non-inferior to the Serum MMR vaccine.

## Introduction

Measles, mumps, and rubella are viral diseases associated with significant morbidity and mortality in children. Measles leads to significant morbidity and mortality in areas of the world where routine vaccination is not practiced, and it is the fifth leading cause of mortality in children aged < 5 years.^1^ Encephalitis is rare, manifesting in approximately 0.1% of patients with measles, but ∼20% of these patients sustain permanent brain damage as a result.^2^ Mumps commonly causes severe forms of meningitis and orchitis.^2^ Infants born to women infected with rubella in their first trimester of pregnancy are at a high risk of congenital rubella syndrome, which may result in death.^3^ Effective vaccination strategies coupled with sustained high vaccination coverage can reduce the risk of such highly infectious diseases.^4^ Combined live attenuated measles, mumps, and rubella (MMR) vaccine became available in the 1970s and helped to increase vaccine coverage against these three viral diseases by improving the convenience of administration and reducing the number of injections a child needed to endure.^2^

M/s Cadila Healthcare Limited, India has developed a novel MMR vaccine (live, freeze-dried) (Cadila MMR vaccine), containing the Edmonston-Zagreb measles strain (≥ 1000 CCID_50_), the Hoshino mumps strain (≥ 5000 CCID_50_), and the RA 27/3 rubella strain (≥ 1000 CCID_50_). The Hoshino strain of mumps has been qualified by the WHO^5^ and is used in various formulations of monovalent mumps vaccines and trivalent MMR vaccines being marketed in Japan, Iran, and other countries for more than 25 years now. The immunogenicity and the safety of the monovalent mumps vaccines and trivalent MMR vaccines with Hoshino mumps strain have been well established over the years.^6-9^ However, this is the first time that the Hoshino mumps strain has been combined with the Edmonston-Zagreb measles strain and the RA 27/3 rubella strain in MMR vaccine.

The Cadila MMR vaccine has been developed in two formulations: a single-dose formulation and a multi-dose formulation (10-dose vial). Both formulations were found safe and immunogenic in preclinical animal studies, phase I studies in adult subjects, and a non-comparative phase II study in pediatric subjects aged 15–18 months (unpublished data).

This phase III clinical trial was conducted to evaluate the immunogenicity and safety of single-dose and multi-dose formulations of the Cadila MMR vaccine, and compare this novel vaccine to the existing MMR vaccine (live, freeze-dried) of M/s Serum Institute of India Limited (Serum MMR vaccine) in healthy pediatric subjects aged 15–18 months. The comparator vaccine in this study, the Serum MMR vaccine, containing the Edmonston-Zagreb measles strain (≥ 1000 CCID_50_), the L-Zagreb mumps strain (≥ 5000 CCID_50_), and the RA 27/3 rubella strain (≥ 1000 CCID_50_), is a WHO pre-qualified vaccine, and is one of the most commonly used MMR vaccines worldwide.

## Results

**Figure 1.**
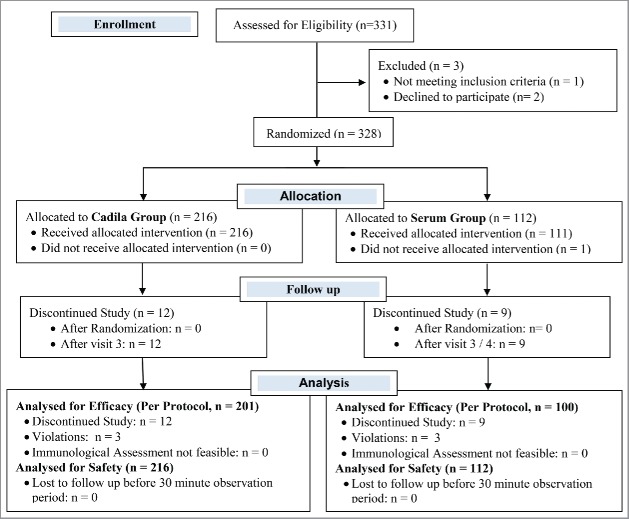
Flow of subjects in the study.

Three hundred and twenty-eight subjects were enrolled in this randomized, single blind, active controlled, and multicenter phase III clinical trial. We assigned 216 subjects to the Cadila group and 112 subjects to the Serum group in a 2:1 ratio. Within the Cadila group, we assigned 108 subjects each to the Cadila single-dose group and the Cadila multi-dose group. All 216 of the Cadila group subjects completed their post vaccination 30-minute observation period and were thus considered for safety analysis. Fifteen Cadila subjects did not complete the study as per the protocol; hence, 201 subjects were considered for the per protocol immunogenicity analysis (101 in the Cadila single-dose group, and 100 in the Cadila multi-dose group). Among the 112 subjects in the Serum group, 100 were considered for the per protocol immunogenicity analysis, and all 112 were considered for safety analysis. The flow of subjects through the study protocol is shown in Fig. 1. The demographic and baseline characteristics of the subjects are shown in Table 1.

**Table 1.** Demographic and baseline characteristics of the enrolled subjects.

	Cadila group	Serum group
	(N = 216)	(N = 112)
Age (Months)	16.4 ± 1.2	16.2 ± 1.2
	(16.2 – 16.5)	(16.0 – 16.5)
Sex^*^		
Male	116 (53.7)	53 (47.3)
Female	100 (46.3)	59 (52.7)
Height (cm)	74.7 ± 4.9	74.0 ± 4.9
	(74.0 – 75.3)	(73.0 – 74.9)
Weight (kg)	9.3 ± 1.3	9.1 ± 1.1
	(9.1 – 9.5)	(8.9 – 9.3)

Data expressed as mean ± SD (95% CI)

* Data expressed as n (%)

### Immunogenicity

The primary immunogenicity end point was the proportion of subjects seropositive at the end of the study. At the end of this study, among the 201 subjects in the Cadila group and the 100 subjects in the Serum group considered for per protocol immunogenicity analysis, all 201 subjects (100.0%) of the Cadila group and 100 subjects (100.0%) of the Serum group were seropositive for anti-measles IgG antibodies, 190 subjects (94.5%) of the Cadila group and 94 subjects (94.0%) of the Serum group were seropositive for anti-mumps IgG antibodies, and 192 subjects (95.5%) of the Cadila group and 91 subjects (91.0%) of the Serum group were seropositive for anti-rubella antibodies (Table 2). The lower limits of the 95% confidence intervals of differences in the proportions of subjects (Cadila group–Serum group) seropositive at the end of the study were 0.0%, −5.0%, and −1.2% for anti-measles, anti-mumps, and anti-rubella IgG antibodies, respectively. These differences exceed the acceptable non-inferiority margin of −10%, thus establishing the non-inferiority of the Cadila MMR vaccine relative to the Serum MMR vaccine.

**Table 2.** Proportion of subjects seropositive at baseline and end of study and the seroconversion rate.

	Cadila group	Serum group	
	(N = 201)	(N = 100)	Cadila – Serum^*^
Measles (cut-off: 200 mIU/mL)^#^			
Subjects seropositive at baseline, n (%)	144 (71.6%)	70 (70.0%)	NA
Subjects seropositive at end of study, n (%)	201 (100.0%)	100 (100.0%)	0.0%(0.0% – 0.0%)
Seroconversion rate, X/Y (%SC)	57 / 57(100.0%)	30 / 30(100.0%)	0.0%(0.0% – 0.0%)
Mumps (cut-off: 8 EU/mL)^#^			
Subjects seropositive at baseline, n (%)	18 (9.0%)	10 (10.0%)	NA
Subjects seropositive at end of study, n (%)	190 (94.5%)	94 (94.0%)	0.5%(−5.0% – 6.1%)
Seroconversion rate, X/Y (%SC)	172 / 183(94.0%)	84 / 90(93.3%)	0.7%(−5.4% – 6.8%)
Rubella (cut-off: 8 IU/mL)^#^			
Subjects seropositive at baseline, n (%)	16 (8.0%)	6 (6.0%)	NA
Subjects seropositive at end of study, n (%)	192 (95.5%)	91 (91.0%)	4.5%(−1.2% – 10.2%)
Seroconversion rate, X/Y (%SC)	176 / 185(95.1%)	86 / 94(91.5%)	3.7%(−2.3% – 9.6%)

N = Total no. of subjects in PP population

n = No. of subjects seropositive

X = No. of subjects seropositive at end of study among those seronegative at baseline

Y = No. of subject seronegative at baseline and considered for calculating seroconversion rate

* Data expressed % (95% CI)

# An antibody titer equal to or greater than the cut-off was defined as seropositive

The secondary immunogenicity end points were the seroconversion rates for measles, mumps, and rubella in subjects who were seronegative before vaccination and the GMTs of anti-measles, anti-mumps, and anti-rubella antibodies in the two groups at the end of the study. Among the subjects seronegative at baseline, the seroconversion rates were 100.0% for measles, 94.0% for mumps, and 95.1% for rubella in the Cadila group, and 100.0% for measles, 93.3% for mumps, and 91.5% for rubella in the Serum group (Table 2). The GMT for anti-measles antibodies at the end of the study was significantly greater in the Cadila group (2355.5 mIU/mL) than in the Serum group (1448.1 mIU/mL) (P < 0.01), while there were no significant differences in the GMTs for anti-mumps antibodies and anti-rubella antibodies (Table 3).

**Table 3.** Post immunization geometric mean titers of anti-measles, anti-mumps and anti-rubella IgG antibodies.

	Post immunization geometric mean titers		
	Cadila group	Serum group	
	(N = 201)	(N = 100)	P Value
Anti-measles antibodies (mIU/mL)	2355.5	1448.1	<0.01
	(2066.9 – 2684.3)	(1223.4 – 1714.0)	
Anti-mumps antibodies (EU/mL)	41.4	52.6	0.20
	(35.4 – 48.4)	(41.0 – 67.6)	
Anti-rubella antibodies (IU/mL)	73.0	53.6	0.75
	(57.0 – 93.5)	(34.7 – 82.8)	

Data expressed as geometric mean (95% CI)

### Sub-group (Cadila single-dose and Cadila multi-dose) analysis

Subgroup analysis of subjects in the Cadila single-dose group and the Cadila multi-dose group showed that there was no significant difference in the proportion of subjects seropositive at the end of the study, or in the seroconversion rate (P > 0.05 for both parameters). Moreover, for all three viruses, both the single-dose and multi-dose formulations of Cadila MMR vaccine were non-inferior to the Serum MMR vaccine in terms of proportion of subjects seropositive at the end of the study, and seroconversion rate (Table 4).

**Table 4.** Sub group (Cadila single-dose and multi-dose) analysis: Proportion of subjects seropositive at baseline and end of study and seroconversion rate.

	CSD	CMD	SII			P value
	(N = 101)	(N = 100)	(N = 100)	CSD – SII*	CMD – SII*	(CMD vs. CSD)
Measles (cut-off: 200 mIU/mL)^#^						
Subjects seropositive at baseline, n (%)	67	77	70	NA	NA	NA
	(66.3%)	(77.0%)	(70.0%)			
Subjects seropositive at end of study, n (%)	101 (100.0%)	100 (100.0%)	100 (100.0%)	0.0%	0.0%	1.00
				(0.0% – 0.0%)	(0.0% – 0.0%)	
Seroconversion rate, X/Y (%SC)	34 / 34	23 / 23	30 / 30	0.0%	0.0%	1.00
	(100.0%)	(100.0%)	(100.0%)	(0.0% – 0.0%)	(0.0% – 0.0%)	
Mumps (cut-off: 8 EU/mL)^#^						
Subjects seropositive at baseline, n (%)	9	9	10	NA	NA	NA
	(8.9%)	(9.0%)	(10.0%)			
Subjects seropositive at end of study, n (%)	93	97	94	−1.9%	3.0%	0.13
	(92.1%)	(97.0%)	(94.0%)	(−8.9% – 5.1%)	(−2.7% – 8.7%)	
Seroconversion rate, X/Y (%SC)	84 / 92	88 / 91	84 / 90	−2.0%	3.4%	0.12
	(91.3%)	(96.7%)	(93.3%)	(−9.8% – 5.7%)	(−3.0% – 9.7%)	
Rubella (cut-off: 8 IU/mL)^#^						
Subjects seropositive at baseline, n (%)	7	9	6	NA	NA	NA
	(6.9%)	(9.0%)	(6.0%)			
Subjects seropositive at end of study, n (%)	94	98	91	2.1%	7.0%	0.09
	(93.1%)	(98.0%)	(91.0%)	(−5.4% – 9.6%)	(0.7% – 13.2%)	
Seroconversion rate, X/Y (%SC)	87 / 94	89 / 91	86 / 94	1.1%	6.3%	0.10
	(92.6%)	(97.8%)	(91.5%)	(−6.7% – 8.8%)	(−0.1% – 12.7%)	

CSD – Cadila Single-dose group; CMD – Cadila multi-dose group; SII – Serum group

N = Total no. of subjects in PP population; n = No. of subjects seropositive; X = No. of subjects seropositive at end of study among those seronegative at baseline; Y = No. of subject seronegative at baseline and considered for calculating seroconversion rate

* Data expressed % (95% CI);

# An antibody titer equal to or greater than the cut-off was defined as seropositive

### Safety

**Figure 2.**
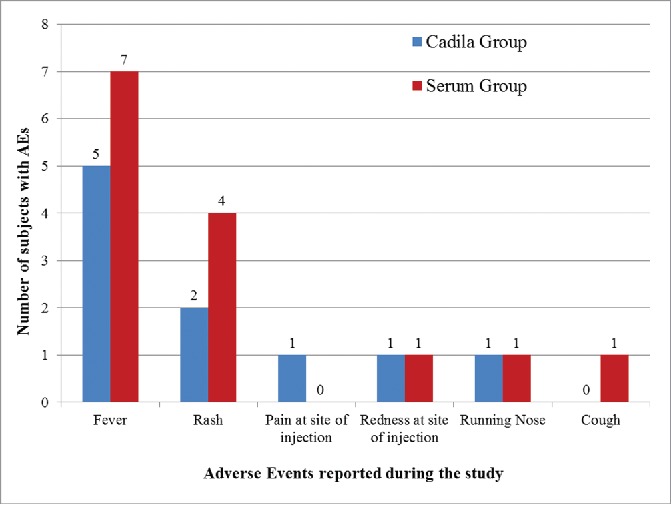
Adverse events reported post MMR vaccination.

Ten adverse events were reported in 7 subjects in the Cadila group (3.2% adverse event rate), and 14 adverse events were reported in 10 subjects in the Serum group (8.9% adverse event rate). The most common adverse event reported during the study was fever in 5 subjects from the Cadila group and 7 subjects from the Serum group, which was followed by rash in 2 and 4 subjects in the Cadila and Serum groups, respectively. There was no difference in the adverse event profile of the two groups (P > 0.05). All adverse events reported in the Cadila group and all adverse events but one (92.9%; fever) reported in the Serum group were ‘Mild’ in severity. Most of the adverse events lasted for 1–3 d (80.0% in the Cadila group, and 92.9% in the Serum group). All of the adverse events resolved completely, with or without symptomatic treatment, during the study period. Among the adverse events reported, 90.0% in the Cadila group and 78.6% in the Serum group were solicited adverse events. No “serious” or “severe” adverse events were reported for the subjects during the study. Among the 10 events in the Cadila group, eight were rated as having a “Possible” association to the study vaccine, one as a “Probable” association, and one was rated “Unrelated” to the study vaccine. Among the 14 events in the Serum group, six had a “Possible” association, three had a “Certain” association, two had a “Probable” association, two were “Unrelated,” and one event had an “Unlikely” association to the study vaccine. Irrespective of their causal relationship to the vaccine, the adverse events recorded during the study are shown in Fig. 2.

## Discussion

This study presents the results of a phase III clinical trial conducted to assess the immunogenicity and safety of single-dose and multi-dose formulations of a novel Cadila MMR vaccine, as compared to Serum MMR vaccine, when administered to healthy pediatric subjects aged 15–18 months, in a population routinely receiving the monovalent measles vaccine at 9 months of age. The Cadila MMR vaccine contains the Edmonston-Zagreb measles strain, the Hoshino mumps strain and the RA 27/3 rubella strain. The Hoshino mumps strain has been combined with AIK-C measles strain and the Takahashi rubella strain in Japan and Iran,^6--9^ but it is the first time that it has been combined with the Edmonston-Zagreb measles strain and the RA 27/3 rubella strain in this novel vaccine. This study is the first to compare an MMR vaccine containing the Hoshino mumps strain with a WHO pre-qualified MMR vaccine.

The immunogenicity results of this study show that the Cadila MMR vaccine is non-inferior to the Serum MMR vaccine with respect to the proportion of subjects seropositive at the end of the study and the seroconversion rates for all three viral strains. Both the single-dose and multi-dose formulations of the Cadila MMR vaccine are similar to each other, and non-inferior to the Serum MMR vaccine. Moreover, the GMTs of both anti-rubella and anti-mumps antibodies are similar in the two groups, but the GMT of anti-measles antibodies is significantly greater in the Cadila group than in the Serum group. This greater rise in the anti-measles antibodies with Cadila MMR vaccine could be attributed to the higher release potency of its measles component (Table 5). However, there could also be a possibility of interference of the L-Zagreb strain of mumps on the immune response of the measles strain. This effect would need to be confirmed in large adequately designed clinical studies.

**Table 5.** Potency of the vaccines used in the study (CCID_50_/dose).

	Serum MMR Vaccine		Cadila MMR Vaccine		
Strains	Strain	Titer	Strain	Titer (SD)	Titer (MD)
Measles	Edmonston–Zagreb	10^3.59^	Edmonston-Zagreb	10^4.09^	10^3.89^
Mumps	L-Zagreb	10^4.48^	Hoshino	10^4.55^	10^4.33^
Rubella	RA 27/3	10^3.82^	RA 27/3	10^3.90^	10^3.90^

SD: Single-dose formulation; MD: Multi-dose formulation

The results obtained in this study with the Cadila MMR vaccine are comparable to those of the various other MMR vaccines available worldwide, although a direct comparison of the results is not feasible owing to the differences in the population studied, strains of the viruses used in the vaccine formulations, time of sampling, and antibody titer cut-offs used for defining seroconversion. In one such previous clinical trial comparing the GSK MMR vaccine with the Merck MMR vaccine, the GMT for anti-measles antibodies was 2342.5 mIU/mL with the GSK MMR and 2548.8 mIU/mL with the Merck MMR vaccine. The seroconversion rates for measles for both the vaccines was 100.0%.^10^ These results of measles in the various MMR vaccines are in line with the results for the Cadila MMR vaccine used in this study.

The GMT of anti-rubella antibodies with the Cadila MMR vaccine (73.0 IU/mL) is also in line with that obtained with the other MMR vaccines in various trials. In one of the trials, the GMT of anti-rubella antibodies obtained with the GSK MMR vaccine was 72.5 IU/mL.^11^ In another trial, the GMT of anti-rubella antibodies obtained with the Merck MMR vaccine was 60.5 IU/mL.^12^ As per the WHO position paper,^13^ all the licensed rubella vaccines induce a seroconversion of approximately 95% after a single dose and up to 5% of the vaccines fail to seroconvert because of concurrent infection or pre-existing maternal rubella antibodies. The seroconversion rate for rubella with the Cadila MMR vaccine in this clinical study (95.1%) is in line with that of the other licensed vaccines.

Although no direct comparison of the seroconversion rates can be made for mumps because of the antibodies not being measured in International Units, the results are similar to those reported previously. In one of the clinical trials evaluating the MMR vaccine of Berna and Merck, the seroconversion rate with the Berna MMR vaccine was 77.4%, while that of the Merck MMR vaccine was 91.3%.^12^ Moreover, as per the WHO position paper^14^ on mumps virus vaccines, various studies in industrialized countries have demonstrated that a single dose of the Jeryl-Lynn strain mumps vaccine results in seroconversion rates of approximately 80–100%, while cumulatively the various vaccine strains of mumps virus (including the Jeryl-Lynn, Urabe, Am9, and Leningrad-3 strains) have achieved seroconversion rates close to 90%. The Cadila MMR vaccine is the first MMR vaccine having the Hoshino strain of mumps virus along with the Edmonston-Zagreb strain of measles and RA 27/3 of strain of rubella. The seroconversion rate obtained with the Hoshino strain of mumps (94.0%) is in line with the seroconversion rate seen with the other strains as mentioned. The immunogenicity results for the Hoshino strain are also in line with the immunogenicity of the earlier MMR vaccine containing the Hoshino strain, wherein the seroconversion rates were reported to be 93.3–96.8%.^6,7^ In addition, the Hoshino mumps strain has been shown to induce cellular immune response in over 90% of recipients, independent of the humoral response; in one study, virus-specific IFN-γ production was observed in 7/12 recipients who did not seroconvert after receipt of an MMR vaccine containing the Hoshino mumps strain.^7^

Although a systematic efficacy study has not been done as the antibody titers and seroconversion rates of the Cadila MMR vaccine are similar to those of the other MMR vaccines used worldwide and meet the WHO requirements, it can be inferred that the Cadila MMR vaccine would be efficacious when used in actual practice.

The safety profile of the Cadila MMR vaccine is found to be similar to that of the Serum MMR vaccine. Both vaccines were well tolerated by the subjects, and no serious adverse events were reported during the study period. In both groups, the most common adverse events reported during the study were fever, followed by rash. The adverse events noted in this clinical trial are in line with the adverse events listed in the package insert of the Serum MMR vaccine (Tresivac of M/s Serum Institute of India Limited) and the published literature.^15^ Although not reported in this clinical study, MMR vaccine containing the L-Zagreb mumps strain is known to be associated with an increased risk of aseptic meningitis,^16,17^ whereas the Hoshino mumps strain has not been associated with any such risk. According to a previous report from Japan, no case of meningitis was reported even after the use of the Hoshino strain in over 700,000 doses in a practical pediatric immunization program.^6^ The safety of the MMR vaccine with the Hoshino mumps strain was also studied in Iran in more than 14,000 subjects aged 12 months and 29,000 subjects aged 4–6 years, and the adverse events following immunization were found to be similar to those of other vaccines.^8^

The results of this phase III clinical trial have shown that the novel Cadila MMR vaccine, wherein the Hoshino mumps strain has been combined with the Edmonston-Zagreb measles strain and the RA 27/3 rubella strain for the first time, is non-inferior to the WHO prequalified Serum MMR vaccine containing the L-Zagreb mumps strain, the Edmonston-Zagreb measles strain, and the RA 27/3 rubella strain. However, a major limitation of this study was its single blind design; the knowledge of the vaccine group to the investigators could have had an impact on the safety assessments. Additional post marketing surveillance will further establish the safety of this M/s Cadila Healthcare Limited MMR vaccine.

## Material and methods

This prospective, randomized, single blind, parallel group, active controlled, multicenter, non-inferiority, phase III clinical trial was conducted at 7 tertiary care centers in India from May 2015 to August 2015. The study was conducted by pediatricians in compliance with the Indian Good Clinical Practice Guidelines, and the Ethical Principles of the Declaration of Helsinki. The study was approved by the Office of the Drug Controller General of India, and was registered with the Clinical Trials Registry of India (www.ctri.nic.in; CTRI/2015/05/005784). The study was initiated after review and approval by the Institutional Ethics Committees at each of the seven participating study centers. Written informed consent was taken from the Legally Acceptable Representative (LAR) of each subject, and the entire process of informed consent was video recorded, as per the regulatory requirements of the country.

### Subjects

We enrolled subjects of either sex, 15–18 months of age, brought to the outpatients department for routine MMR vaccination, whose LARs were willing for the subjects to be followed for 6 weeks after vaccination. Parents or guardians of the subject were required to have adequate literacy to complete the diary cards.

Subjects were excluded from the trial if they had a history of previous measles, mumps, or rubella infection or MMR vaccination, i.e., if they had been exposed to any of these three diseases within 30 d of trial commencement or if they had received measles vaccine less than 3 months prior. Other exclusion criteria included history of anaphylaxis or serious reactions to vaccines, neomycin, gelatin, or albumin; history of convulsions, epilepsy, other central nervous system diseases; severe disease of the haematopoietic system; decompensated heart disease or impaired renal function; an acute febrile illness at the time of randomization; history of serious chronic illness; major congenital defects; immunosuppression (immunosuppressive illness or therapy); any other clinically significant concurrent illness affecting immune response after vaccination; administration of any other parenteral vaccine within 30 d of initiation of the study or planned to be given during the study period; and receipt of blood, blood products, or immunoglobulins during the preceding 3 months. Subjects were permitted to use any medication for the treatment of concomitant diseases or adverse events during the study period that were not known to interact with the immunogenicity of the vaccine. However, a record of the same was maintained in the Case Record Form.

### Study procedures

Subjects satisfying the eligibility criteria were randomized in a 2:1 ratio, as per a centralized computer generated randomization schedule, to receive either the Cadila MMR vaccine or the Serum MMR Vaccine (single-dose formulation). Subjects randomized to the Cadila group were randomized to receive the vaccine from either the single-dose or multi-dose formulation. All the subjects were given 0.5 mL single dose of the vaccine in a single blind manner subcutaneously in the upper arm taking aseptic precautions. The vaccines were reconstituted with water for injection (0.5 mL for single-dose formulation and 5 mL for multi-dose formulation). Only one dose of the vaccine (0.5 mL) was used from the multi-dose formulation, for the purpose of this clinical trial. The potencies of the vaccines used in the study are shown in Table 5. The subjects were monitored for adverse events for at least 30 minutes following vaccination. Thereafter, the subjects were monitored for 42 d on an outpatient basis, with scheduled visits on post-vaccination days 7, 14, and 42.

### Immunogenicity and safety assessments

One milliliter of blood was collected from the subjects before vaccination and 42 d after vaccination for immunogenicity assessments. The serum IgG antibody titers against the measles, mumps, or rubella viruses were assessed by commercial ELISA kits manufactured by IBL International, Germany. The assay cut-offs were as follows: 200 mIU/mL for anti-measles antibodies, 8 EU/mL for anti-mumps antibodies and 8 IU/mL for anti-rubella antibodies. ^18-20^ An antibody titer equal to or greater than the cut-off was defined as seropositive. In initially seronegative subjects, seroconversion was defined as appearance of antibody levels above the cut-off levels. The geometric mean of the post vaccination anti-measles, anti-mumps, and anti-rubella antibody titers was calculated for all subjects considered for immunogenicity analysis at the end of the study.

Diary cards were provided to the parents or guardians of the enrolled subjects to record solicited local adverse events (pain, redness, or swelling) for 7 d and systemic adverse events (fever or rash) for 14 d following vaccination. All other adverse events recorded during the first 2 weeks after vaccination, or any adverse event noted after the first two weeks, were recorded as unsolicited adverse events. The adverse events were graded from grade 1 to grade 3 based on the severity (Table 6). The causality of the adverse events was assessed using the World Health Organization-Uppsala Monitoring Centre (WHO-UMC) criteria.^21^

**Table 6.** Adverse event grading.

Reaction	Mild (Grade 1)	Moderate (Grade 2)	Severe (Grade 3)
Pain	Minor reaction on touch	Cries or protests on touch	Cries when limb is moved or spontaneously painful
Redness / Swelling	< 10 mm	10–30 mm	> 30 mm
Fever	37.5–38.5 °C	38.5–39.5 °C	>39.5 °C
(°C / °F)^*^	99.5–100.4 °F	100.4–103.1 °F	>103.1 °F
Rash / unsolicited AE	Adverse event easily tolerated by the child, causing minimal discomfort and does not interfere with everyday activities	Adverse event sufficiently discomforting to interfere with everyday activities	Adverse event prevents normal everyday activities and requires significant medical intervention

* Axillary temperature

### Statistical analysis

The primary immunogenicity variable was the proportion of subjects who are seropositive at the end of the study. The Cadila MMR vaccine was considered non-inferior to the Serum MMR vaccine if the lower bound of the two-sided 95% CI of the difference between the proportion of subjects seropositive at the end of the study (Cadila group–Serum group) was not less than −10 percentage points. The secondary immunogenicity variables were the Geometric Mean Titer (GMT) at the end of the study and the seroconversion rate. GMTs were calculated by taking the antilog of the mean of the log transformed antibody titers. The unpaired t-test was used to compare the log-transformed data of the antibody titers of the two groups. *P* values < 0.05 were considered statistically significant. Non-inferiority comparison was made for the seroconversion rate as mentioned above.

The data for the Cadila group were sub-grouped into Cadila single-dose and Cadila multi-dose groups. The proportion of subjects seropositive at the end of the study and the seroconversion rates were compared between the two groups using the Chi-square and Fischer's exact tests, and the GMTs were compared using the unpaired t-test. The data from the Cadila single-dose and Cadila multi-dose groups were compared to the Serum group data for non-inferiority.

Immunogenicity assessments were done both for the Per Protocol (PP) Population (defined as all randomized subjects who had completed the trial with no violations, as per the protocol, with both pre- and post-vaccination immunological assessments) and the modified Intention to Treat (mITT) Population (defined as all the randomized subjects with both pre- and post-vaccination immunological assessments, including subjects with protocol violations).

The PP analysis was considered definitive and has been presented in the Results section. The safety population included all subjects who were administered the study vaccine and had been available for a 30 minute observation period for safety assessment.

A sample size of 261 subjects (174 in the Cadila group and 87 in the Serum group) had sufficient power ( > 80%) to demonstrate non-inferiority with a margin of 10%, and an expected responder rate (proportion of subjects seropositive at the end of the study) of 92% for each strain. Considering a dropout rate of 20%, a total of 327 subjects were to be enrolled in the study (218 in the Cadila group and 109 in the Serum group). The 218 in the Cadila group were randomized in a 1:1 ratio to either the Cadila single-dose group or the Cadila multi-dose group.
